# Review and application of group theory to molecular systems biology

**DOI:** 10.1186/1742-4682-8-21

**Published:** 2011-06-22

**Authors:** Edward A Rietman, Robert L Karp, Jack A Tuszynski

**Affiliations:** 1 Department of Cancer Biology, Dana-Farber Cancer Institute, Boston, MA, 02215, USA; 2 Department of Genetics, Harvard Medical School, Boston, MA, 02115, USA; 3 Department of Systems Biology, Harvard Medical School, Boston, MA, 02115, USA; 4 Department of Experimental Oncology, Cross Cancer Institute, 11560 University Avenue, Edmonton, AB, T6G 1Z2, Canada; 5 Department of Physics, University of Alberta, Edmonton, AB, T6G 2G7, Canada; 6 Center of Cancer Systems Biology, St. Elizabeth's Medical Center, Tufts University School of Medicine, Boston, MA, 02135, USA

## Abstract

In this paper we provide a review of selected mathematical ideas that can help us better understand the boundary between living and non-living systems. We focus on group theory and abstract algebra applied to molecular systems biology. Throughout this paper we briefly describe possible open problems. In connection with the genetic code we propose that it may be possible to use perturbation theory to explore the adjacent possibilities in the 64-dimensional space-time manifold of the evolving genome.

With regards to algebraic graph theory, there are several minor open problems we discuss. In relation to network dynamics and groupoid formalism we suggest that the network graph might not be the main focus for understanding the phenotype but rather the phase space of the network dynamics. We show a simple case of a *C*_6 _network and its phase space network. We envision that the molecular network of a cell is actually a complex network of hypercycles and feedback circuits that could be better represented in a higher-dimensional space. We conjecture that targeting nodes in the molecular network that have key roles in the phase space, as revealed by analysis of the automorphism decomposition, might be a better way to drug discovery and treatment of cancer.

## 1. Introduction

In 1944 Erwin Schrödinger published a series of lectures in *What is Life? *[1]. This small book was a major inspiration for a generation of physicists to enter microbiology and biochemistry, with the goal of attempting to define life by means of physics and chemistry. Though an enormous amount of work has been done, our understanding of "Life Itself" [2] is still incomplete. For example, the standard way in which biology textbooks list the necessary characteristics of life--in order to delineate it from nonliving matter--includes metabolism, self-maintenance, duplication involving genetic material and evolution by natural selection. This largely descriptive approach does not address the real complexity of organisms, the dynamical character of ecological systems, or the question of how the phenotype emerges from the genotype (e.g., for disease processes [3]).

The universe can be viewed as a large Riemannian resonator in which evolution takes place through energy dispersal processes and entropy reduction. Life can be thought of as some of the *machinery *the universe uses to diminish energy gradients [4]. This evolution consists of a step-by-step symmetry breaking process, in which the energy density difference relative to the surrounding is diminished. When the universe was formed via the Big Bang 13.7 billion years ago, a series of spontaneous symmetry-breaking events took place, which evolved the uniform quantum vacuum into the heterogenous structure we observe today. In fact the quantum fluctuations of the early universe got blown up to cosmological scales, through a process known as cosmic inflation, and the remnants of these quantum fluctuations can be observed directly in the variation of the cosmic microwave background radiation in different directions. At each stage along the evolution of the universe--from quantum gravity, to fundamental particles, atoms, the first stars, galaxies, planets--there was a further breaking of symmetry. These cosmological, stellar, and atomic particle abstractions can be powerfully expressed in terms of group theory [5].

It also turns out that the very foundation of all of modern physics is based on group theory. There are four fundamental interactions (or forces) in Nature: strong (responsible for the stability of nuclei despite the repulsion of the positively charged protons), weak (manifested in beta-decay), electromagnetic and gravitational. The first three are described by quantum theories: an SU(3) gauge group for the quarks, and an SU(2) × U(1) theory for the unified electro-weak interactions [6-8]. From these theories one can derive, for example, Maxwell's theory of electromagnetism, which is the basis of contemporary electrical engineering and photonics, including laser action. Group theory provides a framework for constructing analogies or models from abstractions, and for the manipulation of those abstractions to design new systems, make new predictions and propose new hypotheses.

The motivation of this paper is to examine an alternative set of mathematical abstractions applied to biology, and in particular systems biology. Symmetry and symmetry breaking play a prominent role in developmental biology, from bilaterians to radially symmetric organisms. Brooks [9], Woese [10] and Cohen [11] have all called for deeper analyses of life by applying new mathematical abstractions to biology. The aim of this paper is not so much to address the hard question raised by Schrödinger, but rather to enlarge the set of mathematical techniques potentially applicable to integrating the massive amounts of data available in the post-genomic era, and indirectly contribute to addressing the hard question. Here we will focus on questions of molecular systems biology using mathematical techniques in the domain of abstract algebra which heretofore have been largely overlooked by researchers. The paper will encompass a review of the literature and also offer some new work. We begin with an introduction to group theory, then review applications to the genetic code, and the cell cycle. The last section explores ideas expanding group theory into contemporary molecular systems biology.

## 2. Introduction to Group Theory

Group theory is a branch of abstract algebra developed to study and manipulate abstract concepts involving symmetry [12]. Before defining group theory in more specific terms, it will help to start with an example of one such abstract concept, a rotation group.

**Figure 1:**
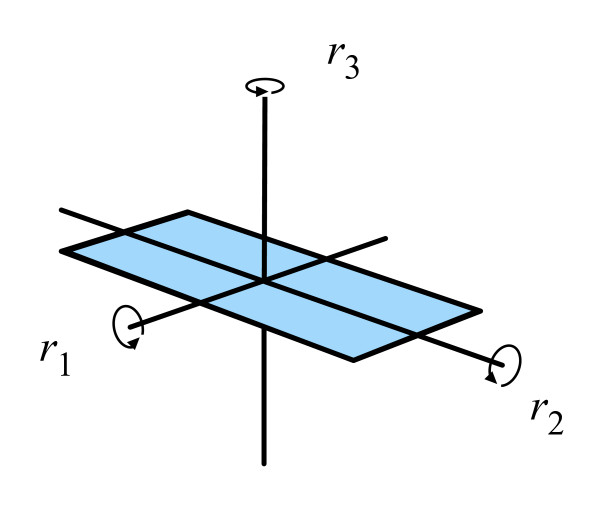
**Card rotations in ℜ^3 ^-space**.

Given a flat square card in real 3-dimensional space (ℜ3-space), we can rotate it π radians, i.e., 180 degrees, around the *X*, *Y *and *Z *axes; let us represent these rotations by (*r*_1_, *r*_2_, *r*_3_) (see Figure 1). We will also assume a do-nothing operation represented by *e*. If we rotate our card by *r*_1 _followed by an *r*_2 _rotation, then we get the equivalent of doing only an *r*_3 _rotation. We can thus fill out a *Cayley *table (also called "multiplication" table, though the operation is not ordinary multiplication). Table 1 shows the full Cayley table for our card rotations in ℜ^3^-space.

**Table 1:** Cayley table for the rotation example (see Figure 1).

	*e*	*r*_1_	*r*_2_	*r*_3_
***e***	*e*	*r*_1_	*r*_2_	*r*_3_

***r_1_***	*r*_1_	*e*	*r*_3_	*r*_2_

***r*_2_**	*r*_2_	*r*_3_	*e*	*r*_1_

***r*_3_**	*r*_3_	*r*_2_	*r*_1_	*e*

The symmetry about the diagonal in the Cayley table tells us that the group is *abelian*: when the rotations are performed in pairs, they are commutative, so that r_m _r_n _= r_n _r_m_.

These four group operations can be written in matrix form as well:

Now we are in position to state the formal definition of a group *G*: it is a nonempty set with a binary operation (denoted here by *) which satisfies the following three conditions:

1. Associativity: for all *a*,*b*,*c *∈ *G*, (a *b) * *c *= a * (b* *c*).

2. Identity: There is an identity element *e *∈ *G*, such that a *e = e* *a *= *a *for all *a *∈ *G*.

3. Inverse: For any *a *∈ *G *there is an element *b *∈ *G *such that a*b = b* *a *= *e*.

Depending on the number of elements in the set *G*, we talk about finite groups and infinite groups. Finite simple groups have been classified; this classification being one of the greatest achievements of 20^th ^century's mathematics. Finite groups also have widespread applications in science, ranging from crystal structures to molecular orbitals, and as detailed below, in systems biology. Among the finite groups the most notable ones are *S_n _*and *Z_n_*, where n is a positive integer. The symmetric group *S_n _*as a set is the collection of permutations of a set of n elements, and has order, i.e., number of elements n!. It turns out that any finite group is the subgroup of a symmetric group for some n. The cyclic group *Z_n _*is a subgroup of *S_n _*consisting of cyclic permutations. *Z_n _*has two other presentations:

1. Rotations by multiples of 2π/*n*.

2. The group of integers module *n*.

These will be discussed later.

Infinite groups are harder to study, but those that have additional structure--like the structure of a topological space or of a manifold--where this additional structure is compatible with the group structure, have also been classified. Of particular interest are the Lie groups, which are simultaneously groups and topological spaces, and the group multiplication and inverse operation are both continuous functions. Lie groups are completely classified, many of them arising as matrix groups. The matrix representation allows us to use conventional matrix algebra to manipulate the group objects, but does not play any special role. In fact any group, finite or infinite, is isomorphic to a subgroup of matrix groups. This is the realm of group representation theory.

The orthogonal groups *O*(*n*) (where *n *is an integer) are made from real orthogonal n by n matrices, i.e., those *n *× *n *matrices *O *for which

The special orthogonal group *SO*(*n*) consists of those orthogonal matrices whose determinant is +1, and they form a subgroup of the orthogonal group: *SO*(*n*) ⊂ *O*(*n*). Geometrically, the special orthogonal group *SO*(*n*) is the group of rotations in n dimensional Euclidian space, while the orthogonal group *O*(*n*) in addition contains the reflections as well.

Similarly, the unitary matrices, *U*(*n*)

form a group (where *H *means complex conjugation of each matrix element together with transposition). Special unitary matrices, *SU*(*n*), satisfy the additional det(*U*) = +1 constraint, and also form groups.

Finally, we mention the "symplectic" or *Sp*(*2n*) groups, but given the fact that these are harder to define, we will not give a formal definition here. As will be shown later, these matrix groups are used in describing the "condensation" of the genetic code.

Another important definition which we will encounter later involves groupoids. A groupoid is more general than a group, and consists of a pair (*G*,*μ*), where *G *is a set of elements, for example, the set of integers *Z*, and *μ *is a binary operation--again usually referred to as "multiplication," but not to be confused with arithmetic multiplication--however, the binary operation *μ *is *not *defined for every pair in *G*. We will see that groupoids are useful in describing networks, and thus transcriptome and interactome networks.

## 3. The Genetic Code

In this section we review some work describing the genetic code in groupoid and group theory terms. One could easily imagine genetic codes based only on RNA or protein, or combinations thereof [13]. When the genetic code "condensed" from the "universe of possibilities" there were many potential symmetry-breaking events.

A codon could be represented as an element in the *direct product *of three identical sets, *S*1 = *S*2 = *S*3 = {*U*, *C*, *A*, *G*}:

The triple cross product has 4^3 ^= 64 possible triplets. As is known, the full three-way product table contains redundancies in the code. This was all worked out in the '60s, without group theory, using empirical knowledge of the molecular structure of the bases [14].

A simple approach to describe the genetic code involves symmetries of the code-doublets. Danckwerts and Neubert [15] used the Klein group; an abelian group with 4 elements, isomorphic to the symmetries of a non-square rectangle in 2-space. The objective is to describe the symmetries of the code-doublets using the Klein group. We can partition the set of dinucleotides into two subsets:

The doublets in *M*_1 _would match with a third base for a triplet that has no influence on the coded amino acid. The doublets in *M*_1 _are associated with the degenerate triplets. Those in *M*_2 _do not code for amino acids without knowledge of the third base in the triplet. Introducing the doublet exchange operators (*e*,*α*,*β*,*γ *) we can perform the following base exchanges:

where the exchange logic is given as follows: *α *exchanges purine bases with non-complementary pyrimidine bases, *β *exchanges complementary bases which can undergo hydrogen bond changes, and *γ *exchanges purine with another purine and pyrimidine with another pyrimidine, and is a composition of *α *with *β*. The operator *e *is our identity operator. The Cayley table for the Klein group is shown in Table 2. The table has the exact form as the rotation table in Table 1 and so they are said to be *isomorphic *with each other.

**Table 2:** Klein group table for genetic code exchange operators.

	*e*	*α*	*β*	*γ*
***e***	*e*	*α*	*β*	*γ*

***α***	*α*	*e*	*γ*	*β*

***β***	*β*	*γ*	*e*	*α*

***γ***	*γ*	*β*	*α*	*e*

**Figure 2:**
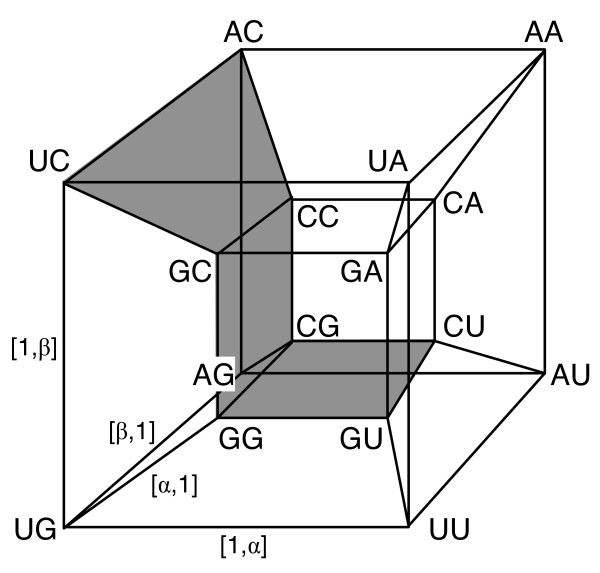
**Doublet genetic code from (*K*4 × *K*4) product**. Figure reproduced after Bertman and Jungck [16].

Bertman and Jungck [16] extended this Klein representation to a Cartesian group product (*K*4 × *K*4), which resulted in a four-dimensional hypercube, known as a tesseract. The corners of the cube are pairs of operators from the Klein group and genetic code for doublets, shown in Figure 2.

The corners of this hypercube form two octets of dinucleotides, the two sets *M*_1 _and *M*_2 _. The vertices of each octet lie at the planes of a continuously connected region. One such region *M*_1 _is shown in the shading of Figure 2. The octets are neither subgroups nor cosets of a subgroup. They are both unchanged under the operations (*e, e*) and (*β*,*e*). These two octets can also be interchanged by acting on one of them with (*α*,α) and/or (*γ*,*α*).

In general, not much can be stated about the product of two groups. If *A *and *B *are subgroups of *K*, then the product may or may not be a subgroup of *K*. Nonetheless, the product of two sets may be very important and leads to the concept of cosets. Let *K *be the Klein group *K *={*e*,*α*,*β*,*γ*} and take the subgroup *H *= {*e*,*β*}, then the set *αH *= {*αe,αβ*} = {*α*,*γ*} is known as a left coset. Since *K *is abelian, the right coset *Hα = *{*eα*,*βα*} = {*α,γ*} and we find *αH *= *Hα*. The following are the four cosets of the (*K*4 × *K*4) genetic exchange operators:

Here, we have written the corresponding dinucleotide next to the operator in the format (*e*,*e*):*AA*, etc.; the bar over some dinucleotides indicates membership in a different octet of completely degenerate codons, while the other dinucleotides are ambiguous codons.

The (*K*4 × *K*4), 4-dimensional hypercube representation in Figure 2 suggests that the 64 elements in the genetic code, the triplets, could be represented by a 64-dimensional hypercube and the symmetry operations in that space would be the codons. Naturally we can form the triple product

to arrive at a 64-dimensional hypercube as the general genetic code. But of course multiple vertices of this hypercube code for the same amino acid. This is said to be a surjective map, because more than one nucleotide triplet codes for the same amino acid. In 1982 Findley et al. [17] describe further symmetry breakdown of the group *D*, and show various isomorphic subgroups including the Klein group and describe alternative coding schemes in this hyperspace.

Above we described the genetic code with respect to inherent symmetries. In 1985 Findley et al. [18] suggested that the 64-dimensional hyperspace, *D*, may be considered as an information space; if one includes time (evolution), then we have a 65-dimensional information-space-time manifold. The existing genetic code evolved on this differentiable manifold, *M *[*X*]. Evolutionary trajectories in this space are postulated to be geodesics in the information-space-time. It should be possible to use statistical methods to compute distances between species (polynucleotide trajectories) by using a metric, say the Euclidean metric:

and from a phylogenetic tree to recreate trajectories in this space. It should be possible to thus see regions of the information-spacetime that have not been explored by evolution. One may speculate on the code-trajectory by bringing in Stuart Kauffman's theory on the adjacent possible [19-21] by a perturbation theory. Further, the curves on this manifold should map, in a complex way, to the symmetry breaking described below, or bifurcation, and thus give a second route to the differential geometry of Findley et al. [18].

**Figure 3:**
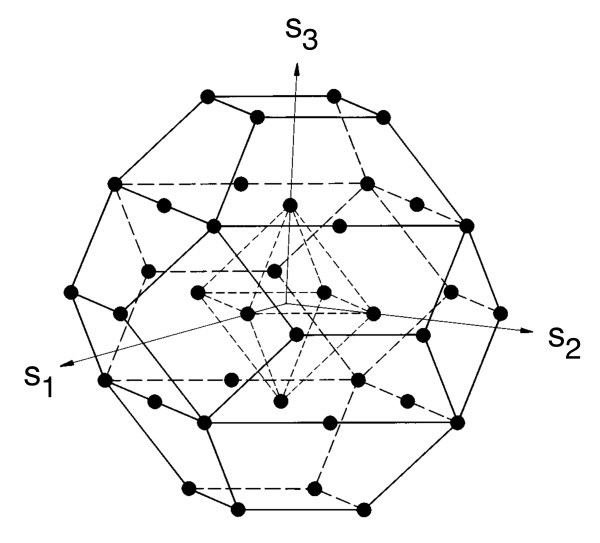
**Weight diagram for *sp*(6)**. Nodes at the central octahedron are four-fold degenerate. Nodes at the centre of the hexagons are two-fold degenerate. Other nodes are non-degenerate. Figure reproduced after Forger et al. [23].

Another approach to understanding the evolution of the genetic code is based on analogies with particle physics and symmetry breaking from higher-dimensional space. Hornos and Hornos [22] and Forger et al. [23] use group theory to describe the evolution of the genetic code from a higher-dimensional space. Technically, they propose a dynamical system algebra or Lie algebra [24]--the Lie algebra is a structure carried by the tangent space at the identity element of a Lie group. Starting with the *sp*(6) Lie algebra, shown in Figure 3, the following chain of symmetry breaking will result in the existing genetic code with its redundancies:

The initial *sp*(6) symmetry breaks into 6 subspaces *sp*(4) and *su*(2). *Sp*(4) then splits into *su*(2) ⊗ *su*(2) while the second *su*(2) factors into *u*(1). Details are given in Hornos and Hornos [22] and Forger et al. [23] on how this maps to the existing genetic code.

## 4. Cell Cycle and Multi-Nucleated Cells

Cell cycle is an example of a natural application of group theory because of the cyclic symmetry governing the process. The steps in the cell cycle include G1 → S → G2 →M, and back to G1. In some cases G0 is essentially so brief as to be nonexistent so we will ignore that state.

To cast the cell cycle into group theory terms recall the definition of a group we gave earlier [25]. The only reasonable approach for casting the cell cycle into group theory is to use the symmetries of a square. Table 3 shows the group table for the cell cycle. It is Abelian and isomorphic to the cyclic group *Z*_4_. Writing the rotation operations for the cell cycle as permutations we get:

**Table 3:** Group table for the cell cycle.

	G1	S	G2	M
**G1**	G1	S	G2	M

**S**	S	G2	M	G1

**G2**	G2	M	G1	S

**M**	M	G1	S	G2

**Figure 4:**
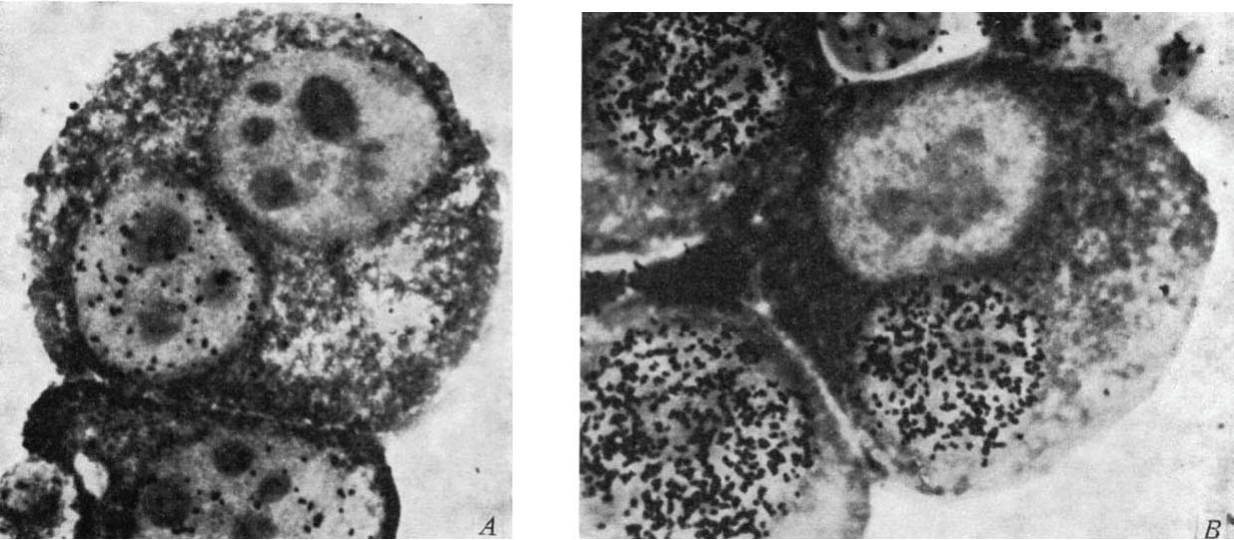
**Photomicrographs of binucleated HeLa cells**. Panel A: A heterophasic S/G2 binucleated HeLa cell at *t *= 0 hours after fusion. Panel B: A heterophasic S/G2 binucleated HeLa cell at *t *= 6 hours after fusion and incubation with ^3^H-thymidine. Figure reproduced after Rao and Johnson [26].

This is isomorphic to *C*4 and *Z*4 cyclic group.

where for example *R*_90 _can be expressed as the mapping:

The cell cycle group table suggests exploring the group operations of some actual physical manipulation of cells. Rao and Johnson [26] and Johnson and Rao [27] conducted experiments on transferring nuclei from one cell into another to produce cells with multiple nuclei. An interesting question they addressed was what effects would a G2 nucleus have when transplanted into a cell whose nucleus was in the S phase? Figure 4 shows an example of a multi-nucleated cell from one of their cell fusion experiments. These experiments were designed to address larger questions about chromosome condensation and the regulation of DNA synthesis.

Some of the nuclei were pre-labeled with ^3^H-thymidine to enhance visibility. Details of the experiments and the results can be found in the original papers. Here we examine, by means of a group table, the converged state for these binucleated cells. Naturally it takes some time for the "reactions" (or not) to take place and for the cell to settle to some stable attractor. In some cases more than one nucleus was added to a cell in another state. For example two G1 nuclei were added to a cell in the S phase. Rao and Johnson [26] and Johnson and Rao [27] recorded the speed to convergence. The group table in Table 4 shows the converged cell state. For example, if a G2 nucleus was added to a cell in G1, there was essentially no change. These are just rough observations; given enough time, all cells will converge to state M, the strongest attractor in the dynamics of the cell cycle. To show that this follows actual group definitions we need to show associativity and find an identity and inverse element, or, alternatively, to show an isomorphism with a known group.

**Table 4:** Group table for the converged stated of binucleated cells (see Figure 4).

	G1	S	G2	M
**G1**	G1	S	G1/G2	M

**S**	S	S	S	M

**G2**	G1/G2	S	G2	M

**M**	M	M	M	M

The table shows that the group is Abelian--that commutativity always holds: *a *◦ *b *= *b *◦ *a *for all *a, b *∈ *G*, where *G *is the group. We can also show associativity, *a *◦ (*b *◦ *c*) = (*a *◦ *b*) ◦ *c*; for example:

and

On the other hand, it is clear from the multiplication table the we cannot have a group structure on the set {G1, G2, S, M}. Namely, in a group *G *any row or column of the multiplication table will contain the elements of *G *precisely once, hence will be a permutation of elements. This property fails for the rows of S and M. Furthermore, the product of G1 and G2 is undefined. Nevertheless, the set {G1, G2, S, M} carries the structure of a groupoid--which is discussed below.

Similar considerations apply if we fuse cells of different type, or differentiation state. These types of experiments were carried out for different stem cells, as reviewed in Hanna [28]. Another fusion-type experiment involves nuclear transfer from one type of somatic cell to another, and determining the identity of the outcome. A variant of this is to transfer RNA populations between cells, and observe the change in the cell's phenotype [29].

## 5. Algebraic Graph Theory: Graph Morphisms

**Figure 5:**
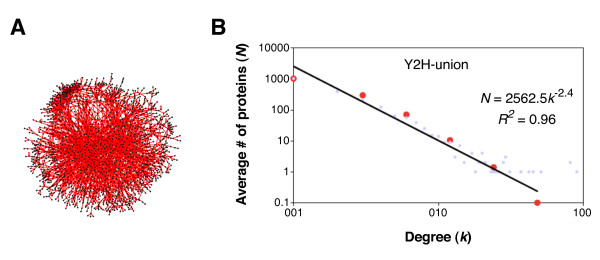
**Protein-protein interaction network and the degree distribution plot**. Panel A: Protein-protein interaction network for the yeast *S. cerevisiae*. Panel B: The degree distribution plot showing a power law behavior. Figure reproduced after Yu et al. [81].

Network graph theory is increasingly being used as the primary analysis tool for systems biology [30,31], and graphs, like the yeast protein-protein interaction (PPI) network shown in Figure 5, are becoming increasingly important. Two excellent references on network theory and network statistics are Newman et al. [32] and Albert and Barabasi [33]. Godsil and Royle [34] and Chung [35] are good references that go beyond the statistical analysis of network graphs and explore mappings from graph to graph, or morphisms and homomorphisms.

With modern datasets it is possible to begin exploring molecular systems dynamics on a network level by using morphism concepts and algebraic graph theory. For example, using these datasets we may be able to impute missing connections in PPI networks, or build vector-matrix-based models representing the dynamics of changing PPI networks. In other cases we may be able to prove algebraic graph theory concepts using the PPI-data. Our focus here will be to continue exploring the cell cycle by including transcription data and protein-protein interaction data from high-throughput screenings. We will first review a few algebraic graph theorems. Godsil and Royle [34] will be our primary reference for algebraic graph theory.

Mathematically a network is a graph *G = G*(*V*, *E*) of a set of *n *vertices {*V*} (also called nodes), and a set of *e *edges {*E*}, or links. Graphs can be represented using the adjacency matrix *A*. The adjacency matrix of a finite graph on *n *vertices is the *n × n *matrix where the non-diagonal *i-j*th entry *A_ij _*is the number of edges from vertex *i *to vertex *j*, while the diagonal entry *A_ii_*, depending on the convention, is either once or twice the number of edges (loops) from vertex *i *to itself.

The eigenvalues of this matrix, *λ_i_*, can be computed to produce the *spectrum *which is an ordered list of the eigenvalues *λ*_1_,*λ*_2_,...,*λ_n_*. This spectrum has many mathematical properties representative of the network graph, though two graphs may have identical spectra. The adjacency matrix however has other useful properties including the following:

Where *tr*(*A*) represents the trace of the matrix, *n *is the number of edges, and *t *represents the number of triangles in the graph. An excellent review of spectral graph theory is given by Chung [35].

Another important matrix is the incidence matrix, which has some very useful properties. The incidence matrix *B*(*G*) of a graph *G*, is a matrix having one row for each vertex and a column for each edge, with nonzero elements for those node-edge pairs for which the node is an end-node of the edge. This matrix is therefore not square. An interesting property is that if we let *G *be a graph with *n *vertices, *c*_0 _its bipartite connected components, and *B *the incidence matrix of *G*, then its rank is given by *rk*(*B*) *n *- *c*_0_.

**Figure 6:**
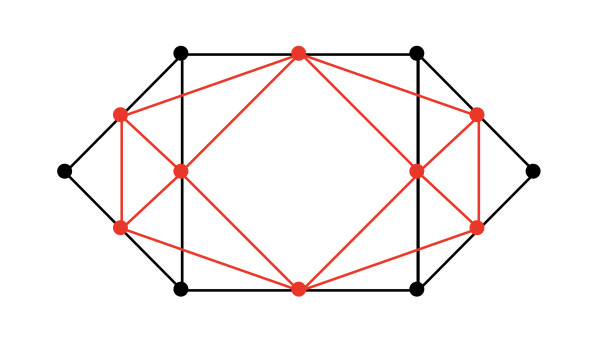
**Example of a line graph**. Diagram obtained from Mathworld [58].

Another observation concerning the incidence matrix involves the line graph of *G*, *L*(*G*). The edges of *G *are the nodes of *L*(*G*), and we connect two vertices with an edge if and only if the corresponding edges of *G *share an endpoint. An example is shown in Figure 6. A theorem proved by Godsil and Royle [34] shows a relation between the adjacency matrix of *L*(*G*) and the incidence matrix of *G*: *B^T^B *= 2*I *- *A*(*L*(*G*)). These simple matrix manipulations allow one to compute potentially new metrics on some complex molecular networks, such as the PPI network in Figure 5.

The concept of automorphism of a graph is an important one, and as we will see it has applicability to subgraphs within more complex graphs. Automorphisms of a graph are permutations of the vertices that preserve the adjacency of the graph, i.e., if (*u, v*) is an edge, and *P *is the graph automorphism, then (*P_u _, P_v_*) is also an edge. As a result, an automorphism maps a vertex of valence *m *to a vertex of valence *m*. Whole graph automorphisms applied to asymmetric graphs, similar to the yeast PPI network shown in Figure 5, detect core symmetric regions.

The automorphisms of a graph forms a group, Aut(*G*). The main question to ask is, what is the size of this automorphism group, represented as |Aut(*G*)|? This provides a measure of the overall network symmetry. Typically, as described by MacArthur and Anderson [36] and Xaio et al. [37], this is normalized for comparing networks of different sizes (*N *is the number of nodes):

MacArthur et al. [38] suggest, and show, that it is possible to decompose, or factor, a large network graph. The NAUTY algorithm [39] they use produces a set known as the automorphism group. The Human B-cell genetic interaction network, for example, can be factored into the terms [40]. The order of this group is computed as

This results from the fact that the order of the cyclic group *C_n _*is *n*--since there are 36 of them we take the 36^th ^power--the order of the symmetric group *S_n _*is *n*!. Given that the network contained 5930 vertices (and 64,645 edges), we have

As a second example MacArthur et al. [38] use data from BioGRID for the *S. cerevisiae *interactome (with 5295 nodes) and obtain the following automorphism group and its order:

*β_G _*in this case is 1.02693.

As we will see later, this may be applicable to molecular interactomes. A full molecular interactome (not just PPI) is a directed graph, and describes an underlying dynamical system in terms of ordinary differential equations: *dx_i _*/*dt *= *f_i _*(*A*,*x_j_*) where *x_i _*is the state of molecular species *i*, and *A *is the full interactome adjacency matrix, an asymmetric matrix. Golubitsky and Stewart [41] point out that the symmetry groups determine the dynamics of the network. When the symmetry changes in one or more factors of the automorphism group, because of a protein mutation or misfolding, for example, this will affect the overall symmetry and thus the dynamics. A catalog of the automorphism groups for interactomes is thus a list of the dynamic behaviors allowed. It might be possible to map these automorphism group elements to disease states. Incidentally, a neural network technique to perform automorphism partitioning is described in Jain and Wysotzki [42].

Another approach to study the dynamics of interactomes exploits a concept known as the Laplacian of the graph [34]. Interactomes are composed of tree-graphs and spanning trees. (The high number of small symmetry subgroups, e.g., , in the automorphism group also indicates this tree topology.) Let *σ *represent an arbitrary orientation of a graph *G*, and let *B *be the incidence matrix of *G^σ^*, then the Laplacian of *G *is *Q*(*G*) = *BB^T ^*. The Laplacian matrix plays a central role in Kirchhoff's matrix tree theorem, which tells us that the number of spanning trees in a *G *can also be calculated from the eigenvalues of *Q*: if *G *has *n *vertices, and (*λ*_1_= 0, *λ*_2_,..., *λ_n_*) are the eigenvalues of the Laplacian of *G*, then the number of spanning trees is given by:

A proof for this theorem is given for example in Godsil and Royle [34].

We can use this theorem to examine the effects of removing a vertex. If we let *e *= *uv *be an edge of *G*, then the graph *G*\*e *is obtained by deleting the edge *e *from *G*. The existing PPI network is an extreme case in which a set of unknown edges *E *and unknown vertices *V *have been removed from the actual interactome to give us the observed graph *P *= *G *\(*E*,*V*).

It would be interesting to see how far these deletion theorems can be extended as one approaches graphs with current density. One should be able to test these new theorems empirically with real world data from a manufacturing plant, say an integrated circuit fab. One could start with the full manufacturome and begin deleting edges or vertices and evaluating the theorems observing the effects on the automorphism groups. We know the full interactome should be a directed graph. With the manufacturome, which is of course a directed graph, it should be possible to evaluate and extend other algebraic graph theorems to directed and undirected graphs.

The last set of theorems we will introduce on algebraic graph theory involves the embedding space or representation of a graph. These theorems are discussed in Godsil and Royle [34]. A *representation ρ *of a graph *G *in ℜ*^m ^*is a map *ρ *: *V*(*G*) → ℜ*^m^*. As an example, a graph with | *V*(*G*) |= 8 and in which each vertex has a valance of 3 can be represented as a cube in 3-space. The center of gravity of the *m*-space object is considered to be the origin for vectors pointing to the vertices. In the case of this example graph, we get:

We say that the mapping is balanced if

where *ρ*(*u*) represents the mapping vectors. We can create a matrix, *R*, of these vectors. The mapping is optimally balanced if and only if, 1*^T ^R *= 0. Usually this will not be the case, especially for complex interactomes and manufacturomes. If the column vectors of *R *are not linearly independent, the image of *G *is contained in a proper subspace of ℜ*^m^*. In this case the mapping *ρ *is just some lower dimensional representation embedded in ℜ*^m^*. The energy of this embedding is found from a Euclidean length:

This suggests it may be possible to asymptotically approach an optimal embedding for nteractomes by a gradient descent algorithm to minimize the energy of the embedding.

A number of questions then arise such as: What is the biological, or evolutionary, significance of the embedding space? How does it relate to the automorphism group and the actual molecular network dynamics? Are patterns noticeable for disease trajectories in this higher-dimensional space, or even simple cell cycle trajectories in this space? Are there routes from differentiated cells to pluripotent states? Are there noticeable automorphism group differences between normal cells and polyploidy cells? Is there an isomorphism between the automorphism group and the motifs of Alon [43], and an isomorphism between the order of the automorphism group | *Aut*(*G*) | and the average degree distribution <*k *> or other network statistics? These are all open research questions and some methods described below may be applicable to efforts aimed at answering these questions.

## 6. Network Dynamics and the Groupoid Formalism

In the above section we described group theory formalism applied to graphs. Here we step up in symmetry, and describe another algebraic object, groupoids; this will allow us to bring more dynamics into the study [41,44,45]. Obviously this has importance for understanding the dynamics of molecular interactome networks.

Recall that a directed graph encodes the dynamics given by *dx_i _/dt *= *f_i _(A*,*x_j_*) where *x_i _*is the state of molecular species *i*, and *A_ij _*is the full interactome adjacency matrix. More precisely the automorphism group of the network implicitly encodes the dynamics. Further, we know that interactome-like network graphs are composed of multiple copies of a few basic components, e.g. . Groupoids are algebraic objects that resemble groups but the conventional group operation is undefined. In other words, we recognize symmetry but automorphisms are nontrivial. This formalism will allow us to apply group-theory methods to network graphs. Most of this will be focused on small subnets within the larger interactome, where we observe permutation-type automorphisms.

The notion of *groupoid *is most transparent if we approach it from a categorical angle [46]. The standard definition of a category ***C ***involves a collection of objects, A, B,..., and a set of morphisms (which could be the empty set) for each pair of objects; Hom(A, B) for objects A and B. The composition of morphisms is defined and is associative, and there is an identity element in each Hom(A, A), therefore Hom(A, A) is never empty.

But a category ***C ***can be viewed as an algebraic structure in itself, endowed with a binary operation, making it similar to a group or semigroup. We call this associated algebraic structure G(***C***). The "elements" (since the collection of objects do not necessarily form a set) of G(***C***) are the *morphism *of ***C***, and the "product" is the composition, which is an associative partial binary operation with identity elements. If ***C ***has only one object, then any two morphisms can be composed, and we have only one identity element. The axioms of a category guarantee that G(***C***) is a semigroup. Furthermore, if we insist on the invertibility of *each *morphism in ***C***, then G(***C***) is a group.

Now it is natural to extend the notion of a group by requiring that the objects of ***C ***form a set, i.e., ***C ***is a small category, and also ask that each morphism of ***C ***is invertible. This is the categorical definition of a groupoid. It is easy to translate this definition into the algebraic language, and get a notion similar to the definition of a group [47]. But perhaps it is the categorical definition that illuminates the power of groupoids. Namely, while groups are ideally suited to describe the symmetries of an object, groupoids can similarly capture the symmetries of collections of objects. This is perfectly illustrated in modern algebraic geometry, when one tries to form classifying space, known as moduli spaces, but the algebraic varieties one wants to classify (say elliptic curves) have different symmetries. This problem is solved using the language of stacks and groupoids [48]. The necessity for the same powerful generalization arises in string theory, where symmetries of the physical theory cannot be mathematically realized in terms of topological spaces and groups, only in terms of stacks and groupoids [49].

In the groupoid approach we will examine not the symmetry of the small subnetworks and motifs, but rather the dynamics of these small networks, when they are directed graphs, and in particular when these small nets are wired together to make larger networks (circuits). The symmetries we will observe are not the network symmetry but the symmetries in the phase space or the space of the dynamics.

The interactome, and indeed the full chemical reaction network comprising a cell is a complicated network with numerous feedback loops and feedforward circuits. Its dynamics is no doubt complicated and the details of the full network are only now being elucidated; but we can begin to speculate on some of the possible dynamics by exploiting work from a slightly more mature field--neuronal networks.

We know that biological neuronal nets comprise two- and three-dimensional arrays of frequency-controlled oscillators, voltage controlled oscillators, and logic gates. Engineers have constructed random and non-random networks of these components and discovered not only that the network is capable of memory storage in the form of dynamic patterns and limit cycles (for example memorizing a Bach minuet) but initially random pulse patterns coursing through the network will, after a time delay for component integration, entrain other components and produce continual limit cycles. In large arrays of these networks the limit cycles interact with each other to produce emergent dynamics. In the following we draw on work of Hasslacher and Tilden [50], Rietman et al. [51] and Rietman and Hillis [52]. We argue that by analogy similar dynamics would occur in molecular interaction network of the cell.

**Figure 7:**
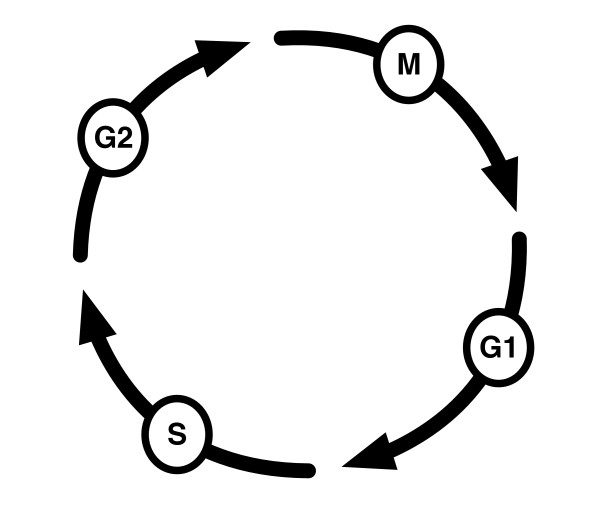
**Schematic of a simplified cell cycle which is isomorphic to *Z*_4_**.

Figure 7 shows a schematic of the cell cycle. As described above, the cyclic group *Z*_4 _is a simple description of the cell cycle, but we can improve the description to incorporate the observation that G1 and G2 are metastable in the same cell. This multi-nucleated state, analogously, could correspond to cancer cells and/or polyploid cells in which we fused the two nuclei. These are also stable, or at least metastable, cell states, and as will be shown below the number of stable states is not huge.

We can let one node in this 4-cycle be represented by the following transfer function in which we include a bias term and its associated Gaussian noise, *θ *+ *ε_θ_*

where *x *is the input signal, and *β *is a gain and can be negative or positive and include noise. The noise is centered about the signal mean and the noise magnitude is set to about one standard deviation of the signal mean. Soft sigmoids have the property of acting like analog signals, not digital. Further, with more than one input feeding into the same node, we sum the product of the incoming signals and their strengths. The transfer function equation now becomes:

Using these dynamics a four-node ring, for example, can exhibit the following three states: (0000), (0101), (0001). Here we employ a permutation-like notation, where, for example (0001) → (0010) → (0100) → (1000) are equivalent to (0001). (Known as the **1 **equivalent class, where the underscore is to remind us that this is not a number but a groupoid.) Interestingly, the three states shown here are isomorphic to the nuclei-fusion group: (0000) → dead cell; (0001) → normal healthy cell; (0101) → G1/G2 (equivalence class **5**).

One can debate whether or not this is a good model of the cell cycle, but feedforward nets of similar central pattern generators (CPGs) are able to rapidly adapt to changing external stimuli to maintain some entrainment or global stability [53], and from a molecular perspective this is exactly what is required of biological cells. The molecular network in living cells consists of a highly complex interconnected feedback and feedforward chemical reaction system. Walhout and colleagues [54,55] and others [56,57] have been discerning some of these details. They have found that feedback and ring circuits, often with inhibitory connections, are common in transcription regulatory networks (protein-DNA interaction networks). One could envision that the basic cell cycle is the primary limit cycle in the dynamics of the cell and the transcription regulator dynamics are used to control and simultaneously be controlled by the cell cycle.

In addition, these ring circuits are able to operate in more than one stable state, exactly as we would need for complex molecular networks of living cells. A 6-node ring circuit can exhibit 5 states; 8-nodes can exhibit 7 states; 10 nodes, 16 states; 12 nodes, 32 states; 14 nodes, 64 states; and 16 nodes 128 states. The increase in states follows a 2-ary necklace function.

where *d_i _*are the divisors of *n *with *d*_1 _= 1, *d*_*v*(*n*) _= *n *; *v*(*n*) is the number of divisors of *n*; ϕ(*n*) is the totient function, and F(.) is the Fibonacci sequence (where F_n _= (F_n-1_) + (F_n-2_) [58]). The totient function, also called the Euler totient function, is the number of positive integers less than *n *which are relatively prime to *n *[51].

Consequently, even small rings of only a dozen nodes can maintain a large number of stable states. Coupling these motifs into networks can produce overall global stability. As Golubitsky and Stewart [41,44] point out--and as is apparent in the large network of Figure 5--the overall network has very low global symmetry.

**Figure 8:**
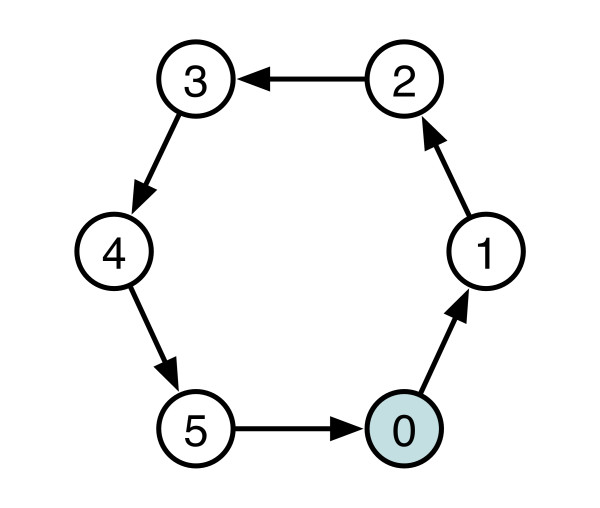
**Diagram of a six-node CPG oscillator**.

To give more details consider the 6-node ring with only one bit active (000001) as a hexagon with one circle filled, as shown in Figure 8. If the active bit is traveling in the counterclockwise direction we can represent the transitioning bit string as follows:

After six-rotations, *r*, the ring dynamics is in the same configuration as when we started. (This is said to be a six-cycle in the terminology of dynamical systems.) Symbolically we can represent this as:

where the numbers are a decimal representation of the bit string; they are underlined to remind us that these are group symbols, and are not to be manipulated as numbers. This string of elements interspersed with a rotation operation represents the elements for the group and the main operation. We represent this group by  where the superscript reminds us that the group is for six-node rings and the subscript is the lowest decimal equivalent of the bit string in this group.

The group  describes only one of the possible cyclic groups within the 6-node ring circuit. Since there are four stable oscillatory states in the 6-node circuit, there are four groups in total. The full set of all the groups  is given as:

The above set of mappings shows cyclic permutations from rotation operations on the individual states *s *represented as decimal equivalent. The  and  groups are said to be of order 6. The  group is of third order and the group  is of second order. The similarities between group theory and conventional dynamics are now obvious. The two 6 order groups are 6-cycles. The one third-order group is a three-cycle and the second-order group is a two-cycle.

The rotation operator (applied once) for each group is different

As the number of rotations needed to return to the starting state decreases for a given group, the periodicity increases--e.g. a two-cycle is faster than a 6-cycle. Similarly, as the number of rotations needed to return to the starting state decreases, the order of the group decreases and the symmetry increases. As we point out later, a symmetry phase transition occurs during signal input and ring coupling.

We can compare this group with conventional cyclic groups. The cyclic group *C*_6 _consists of the decimal numbers {0, 1, 2, 3, 4, 5} and the operation

where *ρ *is the operator that adds two elements in the group *a, b *and then applies the modulus operation. The identity element of *C*_6 _is 0, and the inverse of each element *a *is *b *= 6 - *a*.

The *C*_6 _group table is shown in Table 5. The first row in the table lists the elements of the group. The first column lists the elements of the group, written in the same order as the elements in the first row. The actual arrangements of the elements in the first row/column are not important. The first row is *a *the first column is the element *b*, for the operator *ρ *The elements in the table are generated by the operator, just like a multiplication table.

**Table 5:** The *C*_6 _group table.

	0	1	2	3	4	5
**0**	0	1	2	3	4	5

**1**	1	2	3	4	5	0

**2**	2	3	4	5	0	1

**3**	3	4	5	0	1	2

**4**	4	5	0	1	2	3

**5**	5	0	1	2	3	4

The index *p *of a cyclic group *C_p _*is given by

where *k *| *p *means *k *divides *p*; φ(*k*) is the totient function (as discussed above) and **Z **is the set of integers.

The group table for the  group is given in Table 6. Similar to the group table the elements are written across the first row and first column. Recall the underline is to remind us that these are symbols not numbers. We define the group operation ⊗ according to the following mapping:

**Table 6:** The  group table.

	1	2	4	8	16	32
**1 **	1	2	4	8	16	32

**2 **	2	4	8	16	32	1

**4 **	4	8	16	32	1	2

**8 **	8	16	32	1	2	4

**16 **	16	32	1	2	4	8

**32 **	32	1	2	4	8	16

This maps the CPG group  to the first nonnegative integers in the cyclic group *C*_6 _.

By the defined mapping we have established an isomorphism between these two groups

The other isomorphisms that exist for the *g*^6 ^set of groups are

There are four subgroups in , and there are four subgroups in *C*_6 _:{(0),(0,3),(0,2,4),(0,1,2,3,4,5)}.

In order to use these ideas with concepts such as signal input (known as sensor fusion in the control community) and network (ring) coupling to make larger networks, we need to define operators, Φ*_r_*, that transform one group into another group. Let the subscript on the operator represent the number of rotations when the signal is injected. Then we can write all of the allowed operations on the groups and their results.

To consider sensor input and/or coupling to two or more of these dynamic rings we consider the example . This equation says that when the CPG circuit has one, 1 cycling through the ring and if a pulse of duration equal to the time constant of the nodes is injected at rotation 2 (subscript to operator), this will be the equivalent of initializing the ring circuit with (000101) or decimal 5. Hence, the circuit is transformed to the  group. Explicitly this would be written as (000100) + (000001) → (000101).

As another example consider . This relationship says that when a pulse of two time constants is injected at rotation positions 2 and 3 into a 6-node circuit with a signal already at position 0 (always the assumed initial state), the circuit pulse pattern will transform to . Explicitly this would be written as (000001) + (000110) → (0001001). The other equations are:

These equations are interpreted as follows. From Figure 8 we see a ring in state (000001). If we inject a pulse of short duration (i.e., less then the response time of the logic gates with the associated components) into that ring at position 0 while in that configuration, it will have no effect, . If injected into position 1 while the ring is in this (000001) state it will force the system to transition to state (000101), following the operation . If we inject a short pulse into the network at position 2 it will also transition to (000101) . On the other hand, a short pulse injected at position 3 will cause the ring circuit to exhibit the stable state (001001), according to the operation . In this case, the subscript on the operator indicates the node distance from node 0 in state 1 (000001), while the superscript and subscript on the symbol  remind us that the ring is a 6-node ring and it is in state (001001). These transition rules apply for either injected pulses, such as from external sensors, or for internal pulses, such as from rings coupled to make larger networks. The number of states the ring can sustain is still dictated by the ring size as given by the above 2-ary necklace function.

**Figure 9:**
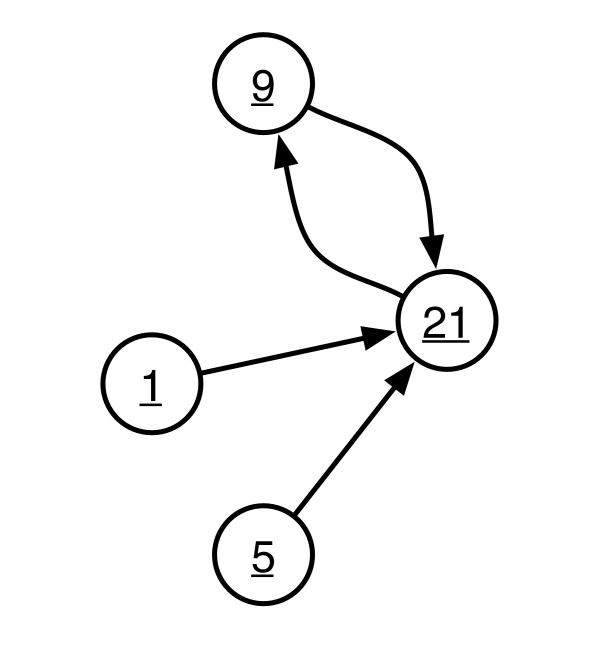
**Attractor diagram corresponding to the circuit shown in Figure 8**.

The significance of this approach is that it describes a global dynamics and entrainment, i.e., a large-scale molecular network dynamics and environmental response, via the dynamics of local internal networks in the interactome. Our concern here is not the symmetry of the interactome, but rather the symmetry of the local and global dynamics. As an example, Figure 9 shows the attractor diagram for the circuit shown in Figure 8. This is a schematic of the dynamics exhibited by the "interactome," the simple feedback circuit of Figure 8. From a group automorphism perspective we can factor the graph in Table 5 to *C*_2 _× *S*_2_, far different from the *C*_6 _network that gives rise to the dynamics shown in Figure 9. This provides an entirely different description of the interactome in terms of the dynamics, rather than in terms of the molecular connectivity. Exploration of this approach to systems biology is an open research issue.

## 7. Cellular Dynamics Models via Graph Morphisms

Our interpretation of the protein-protein interaction (PPI) network, shown in Figure 5, needs to be considered carefully. The first problem it represents is a biophysical interaction of two proteins as observed in yeast 2-hybrid experiments [59,60]. These biophysical interactions do not necessarily occur in the actual organism. Second, the PPI networks for most organisms represent only about 10% of the actual possible protein-protein interactions. Third, it is a static network, or time invariant, which is an almost meaningless concept for life forms. We also know that to include the catalytic set for self-replication, the full interactome should include small molecules, large biopolymers, DNA, RNA, oligobiopolymers, etc.

Given these caveats, we will now proceed to parse the PPI in time. We can do this by conducting a relational join between transcription data and PPI data. We start with the expression data as a function of time. Several expression data sets exist; here we mention only the more recent ones by Pramila et al. [61] and Granovskaia et al. [62]. Both of these teams conducted experiments collecting transcription microarray data at five-minute intervals for the yeast *S. cerevisiae*. The Pramila data (accession number GSE4987) was from cDNA-spotted arrays, and therefore consists of data in the range (-2,2), where zero represents not expressed, below zero represents down regulated, and above zero, up regulated, respectively. The Granovskaia data consist of Affymetrix RNA data (PN 520055) and the numerical data are in the range (-3,2), where data above zero are considered expressed and those below zero not expressed, while the discrimination between up regulated and down regulated is not provided.

The Granovskaia data set is described in their technical paper [62]. They distribute, via links, both the full set of expression data for 6378 gene IDs and a parsed set consisting of 588 genes associated with the cell cycle which clearly show oscillations. Here we ask: What are the large-scale protein-protein interactome changes as a function of time during the cell cycle?

Before we address this question, we note that both the Pramila et al. and the Granovskaia papers show heat maps for the major several hundred genes expressed during the cell cycle. These heat maps show periodic structure and represent periodicity in the transcriptome. Lastly, a paper by de Lichtenbert et al. [63] examines the yeast cell cycle with particular emphasis on parsing the proteome into molecular machines during the cell cycle. Our method differs, as our emphasis is on graph morphisms.

**Figure 10:**
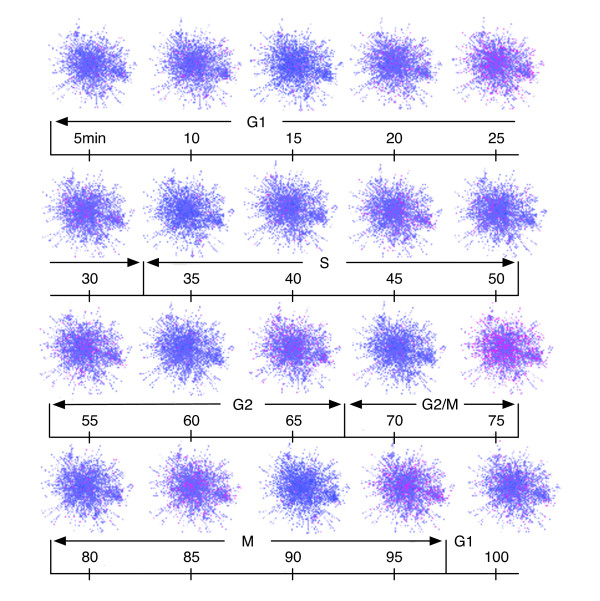
**Yeast protein-protein interactome network changes in 5 minute intervals during the cell cycle**.

The state of the cell at any given point in time is given by the function *x*(*t*). As pointed out above, the transcriptome and the protein-protein interactome (see Figure 5) can be combined to give us a view of the proteins and their connectivity as a function of time, based on the fact that the transcriptome codes for the proteome. Figure 10 shows this mapping relation.

This figure shows for the first time some of the interactome details as a function of time. Each graph represents the changes in the interactome, as represented by the transcriptome, during the indicated time period. Each time period is a 5-minute segment. The red nodes represent those proteins whose expression has disappeared in the time period, and the blue nodes represent those proteins whose expression has appeared in that time period. In a later publication we will be analyzing these graphs in more detail, along with the graph statistical metrics and the automorphism group.

We close this section with a derivation of the matrix *A_ij _*mapping from time-point to time-point--essentially our graph morphism. This matrix can be found by inducing it from the transcriptome data. Recall the transcriptome data *x*(*t*) represents the state change from time-point to time-point. We can use a neural network to induce the matrix *A_ij _*(which is actually two matrices, *A*1 and *A*2) as follows. The mapping is given by

where • represents the product of a matrix with a vector. Here we are using the hyperbolic tangent function, a well-behaved sigmoidal function often used in neural network mappings [64,65]. While these two matrices can be found by the so-called delta rule [65], essentially a gradient descent algorithm [64], we will instead use an extended algorithm cited by Vapnik [66] among others [67]. The cost function for the error minimization is:

where ||*A*|| represents the norm of the sum of the two matrices and *γ *is a Lagrange multiplier called the regularization coefficient. The first term on the RHS is the least mean square of the difference between the target, *T*, and the learning machine response, *R*. This regularization technique effectively forces the values for the adjustable parameters in the nonlinear fit, the weight matrices, to very small numerical values, often near zero. Their magnitude is proportional to the regularization coefficient.

An intuitive argument for this regularization may be found in the analogy of fitting 40 data points to a 6000-order polynomial in 2-space. With 6000 adjustable parameters and using a conventional polynomial-fitting algorithm, the plot of the function with the fitted data points would show wild oscillations in the function, with every data point perfectly intercepted by the function. If we fit the same 40 data points to a third-order polynomial, we would find that many of the points were not intercepted by the curve, and there would be an error associated with the fitting. But comparing interpolation on this third-order polynomial and the 6000-dimensional polynomial, we find that our interpolated errors are much lower and the interpolation is more reliable. Now if we again fit our 40 data points to a 6000-dimensional polynomial, but we also force the magnitude of any of the coefficients to be very small, the net effect will resemble a low-order polynomial. There will be an error associated with the fitting, and a much lower error associated with the interpolation. The regularization algorithm does much the same thing; it forces the magnitude of the weight terms to be small, even very small [66,67].

**Figure 11:**
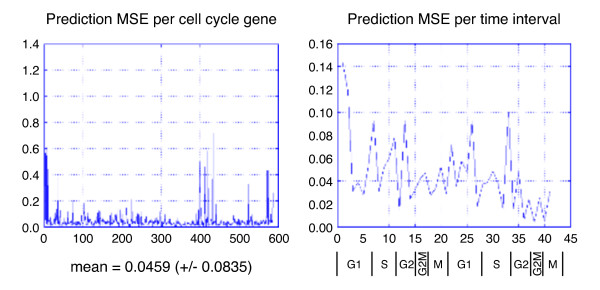
**Prediction error per gene and per time interval**. The x-axis in the gene plot corresponds to the gene ID sequence given by Granovskaia et al. [62]. The x-axis on the time interval consists of 5 minute segments, so value 5 is 25 minutes, etc.

Using the Granovskaia et al. [62] dataset and the 587 genes they identified as relevant to cell cycle, we first made the naïve assumption that the state of the cell, as represented by the transcription data at time *t *would be the same as at time *t *+ 1, with this assumption the mean square error was 0.26. We next carried out the neural network analysis with yeast cell cycle data. We used leave-one-out cross validation to produce the final results. The average mean square error (MSE) from all outputs (all genes) across all time points (41 time points at 5 minute intervals) was 0.0459 (± 0.0835). Figure 11 shows the MSE per gene and the MSE per time interval for prediction from the learning machine. Table 7 shows a table listing all the cell cycle genes with an error > 2 times the standard deviation, 0.1670.

**Table 7:** Table of the gene IDs with error greater than 2 standard deviations (2 × 0

gene ID	**seq. no**.	mse
YKL164C	2	0.5977

YNL327W	5	1.0649

YNR067C	8	0.6106

YOR264W	10	0.4415

YKR077W	36	0.3798

YDR146C	397	0.2492

YGR108W	399	0.5018

YMR001CA	412	0.4618

YJL051W	420	0.5781

YHL028W	433	0.7127

YOR049C	524	0.3274

YPL158C	571	0.4270

YDL179W	575	0.4269

YOL101C	587	0.2593

**Figure 12:**
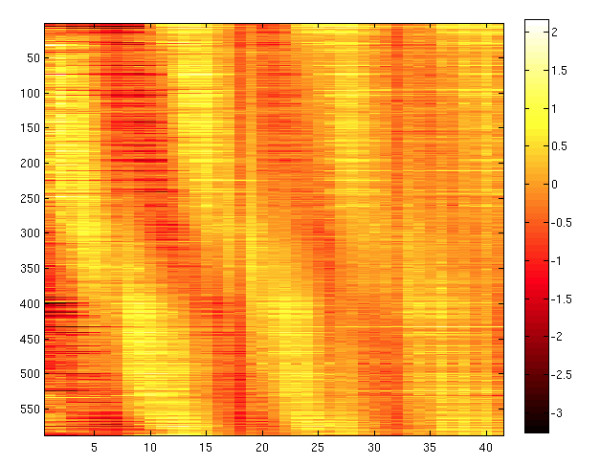
**Expression heat map of the cell cycle**.

Figure 12 shows a heat map plot of expression values for the cell cycle genes as a function of time. The large errors shown in Figure 11 for some of the genes can be explained as expression noise. As shown in the heat map, as time increases the phase in the expression begins to disperse. This is likely due to the phase divergence in the growth of the population and transcription noise.

It should be possible to build a more accurate learning machine for the cell cycle by using a multi-output support vector regression machine [66] or a kernel adatron [68]. In either case the sensitivity analysis is directly computable from the weight matrices for the learning machine. For example, for a multi-output neural network the partial derivative of an output with respect to an input is given by:

With knowledge of the sensitivity analysis we can plot a Pareto chart showing the importance of each of the individual inputs with respect to the output. One could imagine also conducting multi-way digital knockout experiments with this system and comparing it with known experimental results.

## Conclusions

In this review we have touched on a few mathematical ideas that may expand our understanding of the boundary between living and non-living systems. We recognize that there are other important works, including category theory [2,69], genetic networks [70], complexity theory and self-organization [20,69-71], autopoiesis [72], Turing machines and information theory [73], and many others. It would take a full-length book to review the many subjects that already come into play in discussing the boundaries between living and nonliving. Here we focused on mostly group theory and abstract algebra applied to molecular systems biology. Throughout this paper we have briefly described possible open problems. Here we collect them with respect to the subsections of the paper.

In the section on the genetic code we proposed that it may be possible to use perturbation theory to explore the adjacent possibilities in the 65-dimensional space-time manifold of the evolving genome. One could start by using phylogenetic mappings as historical data on this manifold and compute distances in this space. The statistics of these distances may then be fed back via the perturbation theory to study the trajectory. Of course, we recognize that the existing state-of-the-art bioinformatics makes this proposal mostly unfeasible at this time. But crude outlines of the technique could be developed.

With regards to algebraic graph theory, there are several minor open problems we discussed. Here we reiterate only a few. First, it may be possible to map the automorphism group to disease state through an isomorphism with the phase space. Second, it may be possible to use some of the graph deletion concepts *G *\(*E*,*V*) to evaluate existing protein-protein interactomes. One would start with a known complete graph, say a manufacturing plant, remove edges (vertices) and compute conventional network graph statistics. Third, we pose the following questions. What is the minimal embedding space for something like a protein-protein network? Are there patterns in that space? What biological significance is there to these observed patterns?

In the section on network dynamics and groupoid formalism we suggested that the network graph might not be the main focus for understanding the phenotype but rather the phase space of the network dynamics. We showed a simple case of a *C*_6 _network and its phase space network. We envision that the molecular network of a cell is actually a complex network of hypercycles and feedback circuits that could be better represented in a higher-dimensional space. Targeting one protein may not have much effect on the overall phenotype or the overall phase space dynamics. For example, a planar array of 6-cycles would give rise to frustration points, not unlike a spin-glass [74]. The overall dynamics would then give rise to not only emergent phase space dynamics but also emergent patterns in the phase space that would not be computable from the molecular reaction network graph [75-79]. Targeting one or two proteins in this network, based on molecular interaction maps, may prove to be futile in many cases. We conjecture that targeting nodes in the molecular network that play key roles in the phase space, as revealed by analysis of the automorphism decomposition might be a better way to carry out drug discovery and treatment of cancer [80].

## Competing interests

The authors declare that they have no competing interests.

## Authors' contributions

EAR did the research, conceived of the idea of a review paper on the uses of group theory in systems biology, provided most of the material presented in this paper and wrote the first draft. RLK corrected much of the group theory material and made extensive edits of the manuscript. JAT assisted in presenting and integrating the material into the manuscript and coordinated the project. All authors read and approved the final manuscript.
